# Analysis of recurrence and metastasis patterns and prognosis after complete resection of retroperitoneal liposarcoma

**DOI:** 10.3389/fonc.2023.1273169

**Published:** 2023-12-22

**Authors:** Xiaofeng Gao, Pingan Ding, Zhidong Zhang, Yong Li, Qun Zhao, Dong Wang, Xuefeng Zhao, Yu Liu, Bibo Tan

**Affiliations:** Hebei Cancer Clinical Medical Research Center, The Fourth Hospital of Hebei Medical University, Shijiazhuang, China

**Keywords:** retroperitoneal liposarcoma, complete resection, prognosis, recurrence, metastasis

## Abstract

**Objective:**

To analyze the recurrence and metastasis patterns and prognosis after complete resection of retroperitoneal liposarcoma.

**Methods:**

The clinical postoperative follow-up data and results of patients who underwent complete resection of retroperitoneal liposarcoma from September 10, 2014, to September 8, 2021, at Hebei Medical University hospital were collected retrospectively.

**Results:**

A total of 60 patients with complete resection of retroperitoneal liposarcoma, including 33 cases of retroperitoneal liposarcoma recurrence, 2 cases of liver metastasis, and 1 case of lung metastasis, were included. The results showed that 100% of the recurrent sites were located in the primary region of the tumor, with most recurrences located near the kidney, paracolic sulci, and iliac vessels. Three patients had distant metastasis without obvious recurrence on imaging examination. The pathological type of retroperitoneal liposarcoma, Ki67 expression, and presence of serum albumin were risk factors for recurrence and metastasis after complete resection of retroperitoneal liposarcoma. The malignancy and Ki67 expression were independent risk factors for recurrence and metastasis as well as for overall survival of patients undergoing complete resection of retroperitoneal liposarcoma.

**Conclusion:**

Complete resection remains the most effective method to treat retroperitoneal liposarcoma. Patients with pathological types of retroperitoneal liposarcoma showing dedifferentiation, pleomorphism, mixed type, and high Ki67 expression should be closely monitored and observed after complete resection, especially for imaging changes in the primary tumor area.

## 1 Introduction

Liposarcoma is a rare malignancy derived from mesenchymal cells; retroperitoneal liposarcoma (RPLS), which occurs in the retroperitoneal region and is the most common retroperitoneal tumor, accounts for approximately 1% of all malignancies, 25% of all soft tissue sarcomas, and 45% of primary retroperitoneal soft tissue sarcomas (1–3). RPLS refers to a kind of soft tissue malignant tumor originating from adipose tissue and occurring in the retroperitoneal space. It is rare, with a global prevalence rate of approximately 3–4 cases/million per year (4). Although RPLS can occur at all ages, it is rare in teenagers. RPLS peaks at the age of 40–60 years and is slightly more common in men than in women. RPLS includes different histological types of tumors, accounting for approximately 19% of all liposarcomas (5). RPLS occurs in the wide retroperitoneal space, where it is anatomically deep and relatively obscure. Early diagnosis of RPLS is difficult, and most diagnoses are through physical examination. Small tumors show no obvious clinical symptoms or signs; however, as the tumor grows in size, it often compresses and infiltrates adjacent vital organs, blood vessels, and nerves. Most of the symptoms are caused by tumor compression, and the most common symptoms are nonspecific clinical symptoms such as abdominal pain and distension, changes in stool traits, intraperitoneal effusion, and paresthesia of the lower limbs (6). A large mass can occupy much of the abdomen and pelvis and drive the bowel, easily wrapping around the large blood vessels and abdominal and pelvic organs, and growing aggressively along each tissue space. At present, chemoradiotherapy and targeted therapy have limited efficacy in retroperitoneal tumors, and surgery is still the optimal treatment option (7). Initial complete resection with a negative endoscopic margin (R0) is the surgical goal and key to reducing recurrence.

Currently, surgery is known to be the main treatment for RPLS, but postoperative recurrence is common (8). Even after complete capsulectomy and radical resection of the tumor, a complete cure is rarely achieved, which is an important diagnostic and treatment problem associated with the disease (9). A discussion on the recurrence and metastasis patterns of RPLS is scarce in the literature. Therefore, we retrospectively analyzed the clinical and pathological data of patients after RPLS surgery and statistically analyzed the risk factors, patterns of recurrence and metastasis, and impact on survival outcomes of patients with RPLS who underwent complete resection.

## 2 Methods

### 2.1 Clinical data

Cases of 60 patients with RPLS, who were treated by surgery at Hebei Medical University from September 10, 2014, to September 8, 2021, were analyzed retrospectively. The inclusion criteria were as follows: (1) diagnosed with retroperitoneal tumors preoperatively through imaging examinations such as computed tomography (CT) scans and absence of a history of other malignant tumors (10). In addition, the availability of complete clinical and pathological data as well as postoperative follow-up data of the patients; (2) the retroperitoneal tumor was completely removed by surgery with the naked eye, and the negative surgical resection margin was pathologically confirmed; and (3) liposarcoma was confirmed by pathological examination after the operation. The exclusion criteria were as follows: (1) a history of other malignant tumors and other serious diseases; and (2) incomplete clinical, pathological, and follow-up data.

### 2.2 Methods

#### 2.2.1 Treatment methods

Preoperative imaging, such as CT, confirmed the retroperitoneal tumor and the operation was radical (gross complete resection of R1 including combined organ resection) or pathology confirmed the negative surgical margin. Each patient will undergo regular follow-up examinations after complete resection, with a follow-up period of 1 month, 3 months, 6 months, and 1 year. Dedifferentiated patients will receive adjuvant chemotherapy, with a chemotherapy regimen of pirarubicin combined with amitripramide.

### 2.2.2 Clinical data collection

General clinical data (sex and age), CT image data (tumor size and location), pathological data (resected tumor size and pathological type (high differentiation, dedifferentiation, mucus type, polymorph, and mixed type), tumor margin after radical surgery (gross complete resection, pathological result of surgical margin), and intraoperative data (simple tumor resection and combined organ resection) of patients who underwent complete resection of RPLS included in the study were collected.

### 2.2.3 Follow-up

The patients were followed up by telephone, outpatient re-examination, and examination upon hospitalization. The follow-up examination methods were mainly through CT, magnetic resonance imaging, and other imaging examinations. The follow-up ended on January 1, 2023, with a median follow-up of 54 months.

### 2.3 Statistical analysis

Statistical analysis was performed using SPSS27.0 software. The enumeration data were compared using the χ2 test, expressed as percentage. In this study, we performed a univariate analysis of the factors likely to affect the recurrence and metastasis of RPLS. Multivariate logistic regression was included for variables that significantly differed in the recurrence of RPLS to analyze independent influencing factors for recurrence. The factor analysis of influencing factors of prognosis was performed using the Cox regression model, and the difference with P<0.05 was statistically significant.

## 3 Results

### 3.1 Basic characteristics

Sixty patients (32 men and 28 women), with maximum, minimum, and average ages of 71 years, 26 years, and 50.75 years, respectively, were included in the study, Among them, 18 patients were found to lack clear symptoms during a physical examination. During medical consultation, the main symptoms manifested as abdominal pain in 8 patients, abdominal distension in 16, palpable abdominal mass in 12, changes in stool frequency and traits in 2, lumbago and leg pain in 1, frequent micturition and urgent urination in 1, and gross intermittent hematuria in 1 case. Preoperative CT showed that the minimum, maximum, and average diameters were 4.8 cm, 60 cm, and 21.54 cm, respectively. The presence of a single mass, multiple masses, simple mass resection, and combined organ resection was observed in 40, 20, 40, and 20 patients, respectively. According to abdominal quartering, the primary site was located in 6, 14, 6, and 8 patients in the right upper abdomen, left upper abdomen, right lower abdomen, and left lower abdomen, respectively. In 12 patients, the tumors were located in the left abdomen, both left upper and left lower abdomen; 10 patients had tumors in the right abdomen; and 2 patients had tumors located in the lower abdomen, both left lower and right lower abdomen. The tumors were larger than those in the four abdominal regions where they were distributed.

### 3.2 Recurrence and metastasis patterns of RPLS after complete resection

**Figure 1:**
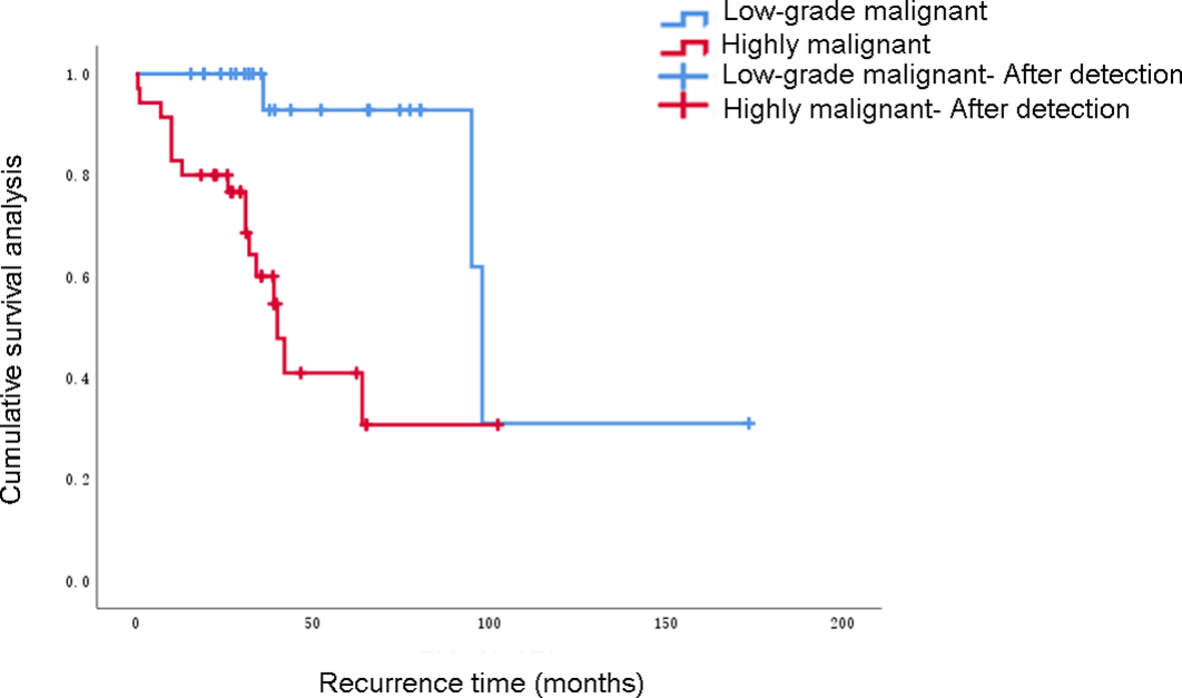
Recurrence and metastasis pattern of retroperitoneal liposarcoma after complete resection.

In this study, 60 patients who underwent complete resection of RPLS were included. According to the abdominal quartering method, the primary site was located in the right upper abdomen, left upper abdomen, right lower abdomen, and left lower abdomen in 6, 14, 6, and 8 patients, respectively. Twelve patients had tumors located in the left abdomen, both left upper and left lower abdomen, and ten patients had tumors in the right abdomen. Two patients had tumors in the lower abdomen where tumors were located in both the left lower and right lower abdomen, and the tumors were larger than those in the four abdominal regions. The primary site was located in the left abdomen (56.7%). There were 33 cases of recurrence, with a recurrence rate of 55% and the recurrence site was 100% located in the primary area. Distant metastasis occurred directly after surgery in three cases at a metastasis rate of 5%, of which liver metastasis was 3.33% and lung metastasis was 1.67%, as shown in **Figure 1**. Fourteen cases underwent more than 2 surgeries, and 2 patients received three-dimensional conformal radiotherapy after recurrence.

### 3.3 Factors affecting recurrence and metastasis of RPLS after complete resection

In this study, 36 of the 60 patients who underwent complete resection of RPLS showed recurrence and metastasis; of these 21 had pathologically dedifferentiated liposarcoma, 5 had mucinous, 3 had mixed, 4 had highly differentiated, and 3 had pleomorphic pathological types. Univariate analysis showed that recurrence and metastasis of RPLS after complete resection were related to the pathological type, Ki67 expression, and serum albumin level but not to sex, age, height, weight, body mass index, hemoglobin level, tumor diameter, single and multiple tumors, or whether the tumor was partitioned or lobulated by CT (**Table 1**). Logistic regression analysis showed that malignancy and Ki67 expression were independent risk factors for the recurrence and metastasis of RPLS after complete resection, as shown in **Table 2**.

**Table 1:** Single-factor analysis of recurrence and metastasis of retroperitoneal liposarcoma after complete resection.

Clinical features	No recurrence (n=24)	Recurrence (n=36)	χ2/t	P
Sex				
Male	12 (50.00%)	20 (55.56%)	0.044	0.833
Female	12 (50.00%)	17 (44.44%)		
Age, y	50.75 (11.51)	55.69 (10.54)	1.716	0.092
Height, cm	165.92 (7.73)	164.97 (7.88)	0.458	0.648
Weight, kg	64.88 (13.16)	66.35 (10.55)	0.483	0.632
BMI	22.87 (4.87)	24.58 (2.87)	1.536	0642
Hemoglobin, g/L	130.42 (21.4)	121.88 (20.86)	0.469	0.130
Albumin, g/L	40.20 (6.07)	33.5 (7.11)	3.733	0.000
Tumor diameter, cm	17.38 (11.06)	21.43 (12.69)	0.831	0.410
Ki67, %	6.505 (9.27)	16.37 (14.82)	3.713	0.002
Pathological type				
Pleomorphic LPS	1 (4.17%)	3 (8.33%)	18.929	0.001
Well-differentiated LPS	12 (50.00%)	4 (11.11%)		
Mixed LPS	0 (0%)	3 (8.33%)		
Myxoid LPS	6 (25.00%)	3 (8.33%)		
Dedifferentiated LPS	5 (20.83%)	23 (63.89%)		
CT imaging				
Single shot	19 (79.17%)	27 (75.00%)	0.140	0.709
Multiple shot	5 (20.83%)	9 (25.00%)		
Surgical resection range				
Simple tumor resection	18 (75%)	21 (58.33%)	1.758	0.185
Combined organ resection	6 (25%)	15 (41.67%)		
Blood transfusion volume	410.00 (1351.86)	768.19 (811.85)	1.283	0.204
Neutrophils/lymphocytes	3.50 (2.50)	4.03 (2.89)	0.724	0.471

LPS, liposarcoma.

**Table 2:** Logistic regression results.

Clinical pathological factors	β	SE	95%CI	P
Albumin	0.720	0.290	0.276	0.860
Ki67A	0.048	0.068	0.918	1.198
Low-grade malignant	0.411	1.456	0.087	26.202
Highly malignant	5.594	2.149	0.000	0.251

### 3.4 Factors affecting the overall survival rate and progression-free survival rate of patients undergoing complete resection of RPLS

**Figure 2:**
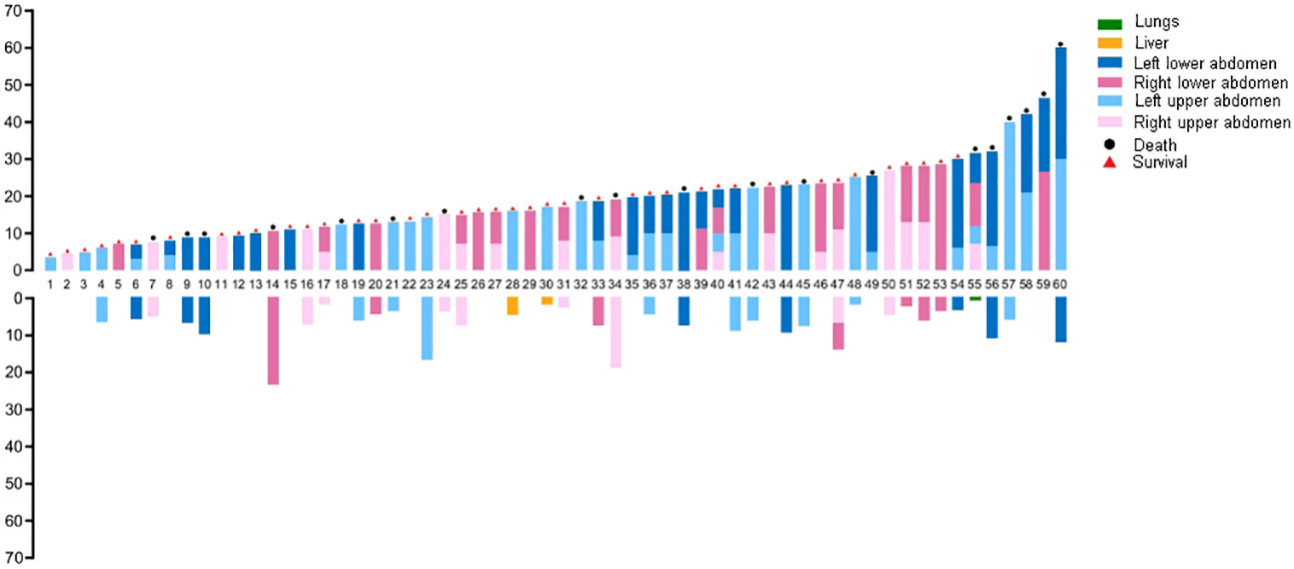
Progression-free survival.

**Figure 3:**
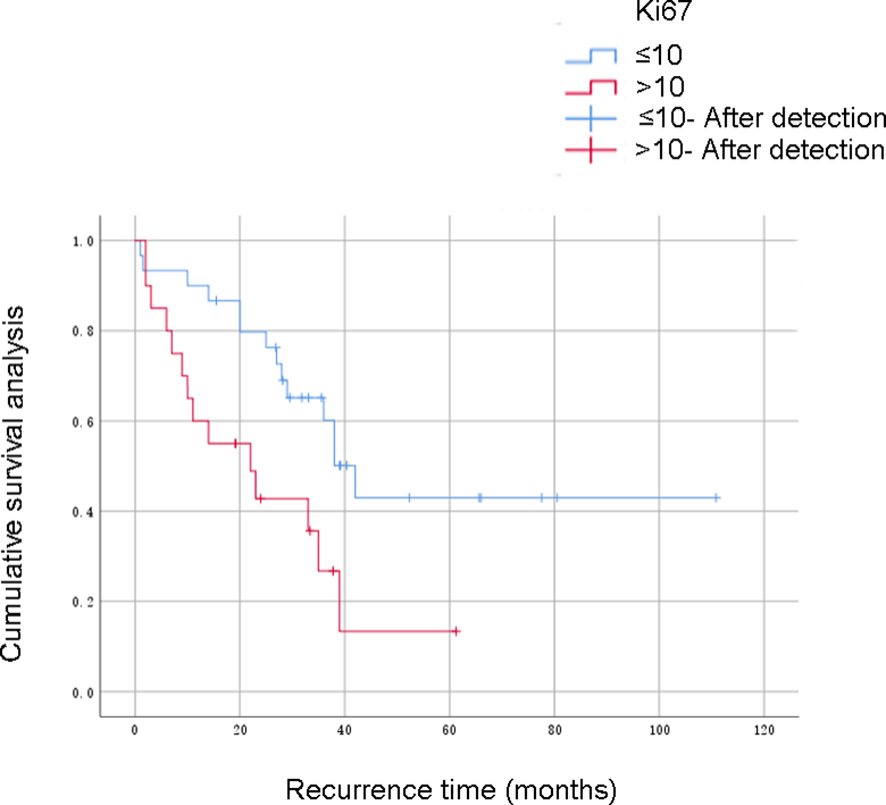
Progression-free survival.

**Figure 4:**
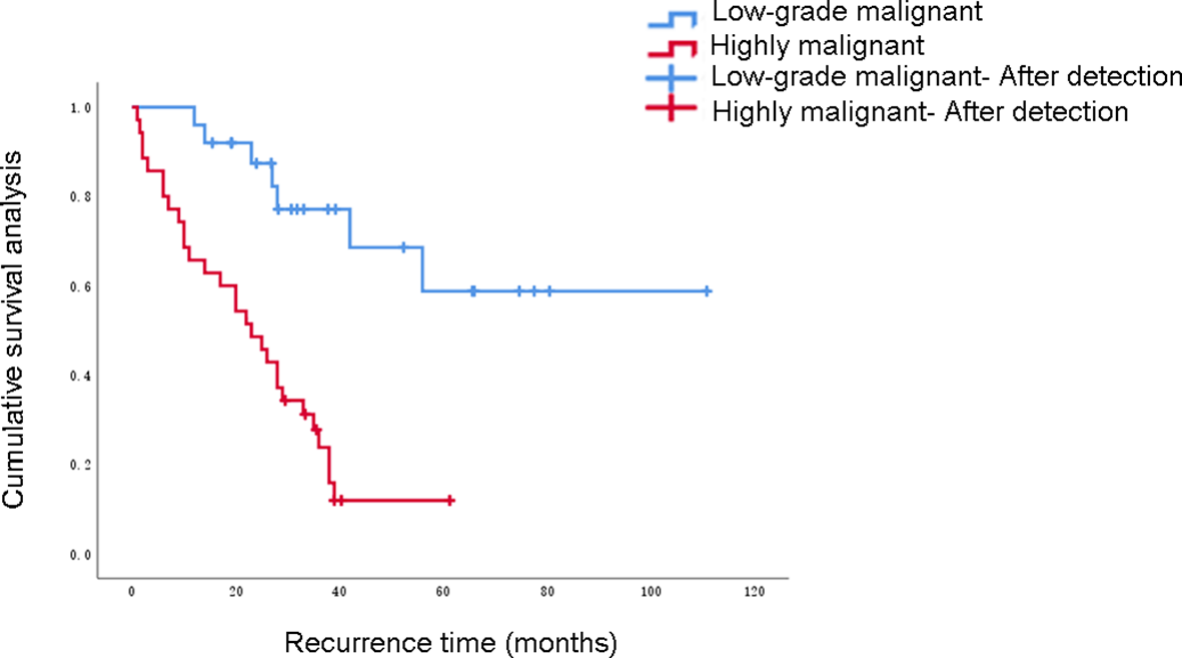
Progression-free survival.

**Figure 5:**
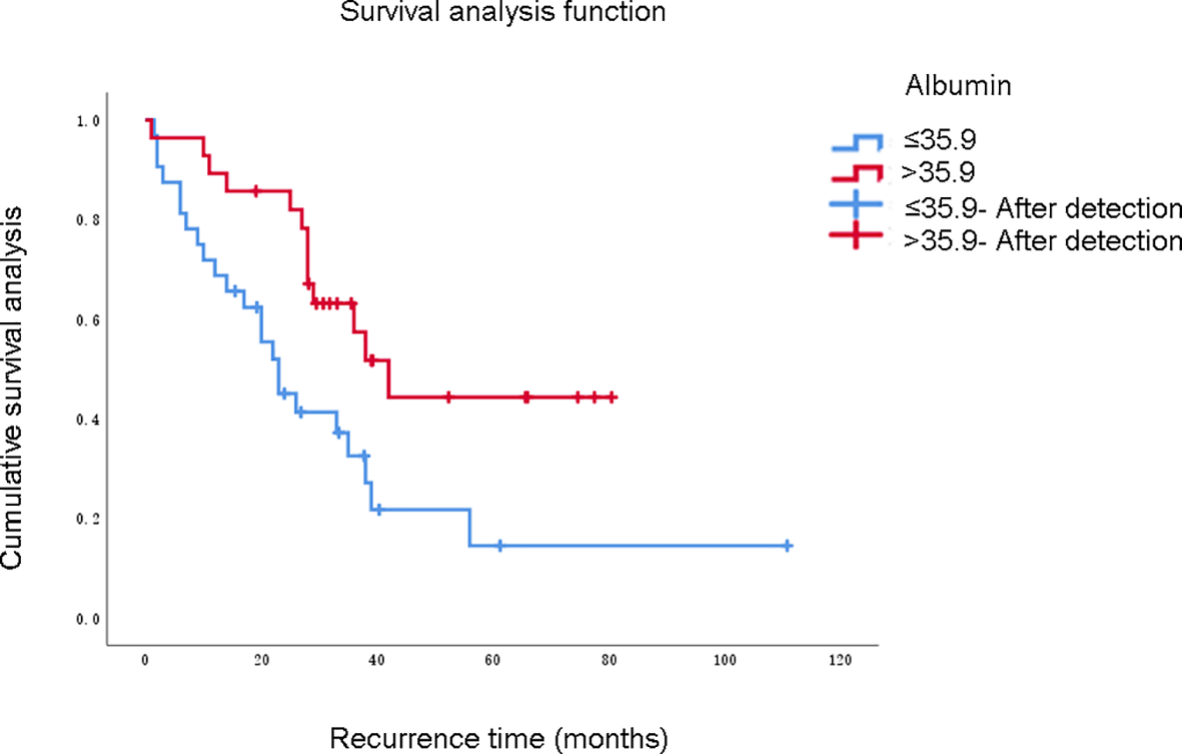
Total survival.

In this study, 60 patients who underwent complete resection of RPLS survived from 0.5 to 196 months as of the follow-up date, and 41 patients were still alive. The 1-, 3- and 5-year OS rates were 91.7%, 71.1%, and 46.6%, respectively. The PFS rates were 76.7%, 54.3%, and 27.8%, respectively. The survival curve was drawn using the Kaplan–Meier method, and the influencing factors were analyzed by the Cox risk regression model. The results showed that the degree of malignancy, Ki67-positivity expression rate, and albumin level were risk factors for PFS in patients undergoing complete resection of RPLS. Among them, the degree of pathological OS factors for progression-free survival of PFS in patients undergoing complete resection of RPLS (**Figures 2**–**4**, **Table 3**). Malignancy and Ki67 expression were also risk factors for OS in patients undergoing complete resection of RPLS (**Figure 5**, **Table 4**).

**Table 3:** Factors influencing disease-free survival rate.

Category	Grouping	Cases	Univariate analysis	Multivariate analysis
Tumor diameter (grouped by median)	<=17	31	0.633	
	>17	29		
Malignancy degree			<0.001	0.001
	High	25		
	Low	35		
Ki67 (grouped by median)	<=10	35	0.002	0.017
	>10	25		
Single shot CT imaging	1	46	0.605	
	2	14		
Surgical resection range	1	39	0.096	
	2	21		
Albumin grouping	<=35.9	32	0.012	0.918
	>35.9	28		

**Table 4:** Factors influencing overall survival rate.

Category	Grouping	Cases	Univariate analysis	Multivariate analysis
Tumor diameter (grouped by median)	<=17	31	0.209	
	>17	29		
Malignancy degree			0.004	0.007
	Low	25		
	High	35		
Ki67 (grouped by median)	<=10	35	0.073	0.223
	>10	25		
Single shot CT imaging	1	46	0.202	
	2	14		
Surgical resection range	1	39	0.063	
	2	21		
Albumin grouping	<=35.9	32	0.071	0.408
	>35.9	28		

## 4 Discussion

Patients with multiple recurrences have progressively shorter recurrence intervals, and approximately 75% of patients die from recurrent tumor recurrence (11), the rate of which has been reported as 55%. According to the abdominal quartering method, 100% of recurrence sites are located in the primary abdominal subregion, mostly near the perinephric region, paracolonic groove, and iliac blood vessels. RPLSs are prone to relapse in situ, which, we believe, may be because of the following reasons: (1) Because most RPLSs have a complete capsule, most surgeons probably consider that it is not aggressive, and aim to ensure the integrity of the capsule during the operation. However, the invasive ability of RPLS may exceed that established previously by the surgical community. The Dana-Farber Institute of Oncology found that the endoscopic infiltration depth of both highly differentiated and poorly differentiated liposarcoma was more severe than that indicated by a macroscopic evaluation during the operation (12). Gronchi et al. found that a positive surgical strategy combined with the resection of organs 1–2 cm around the tumor could significantly reduce the local recurrence rate (61–36%) (13). In addition, different histological types of RPLS have different biological characteristics and different invasive abilities, which are related to their recurrence (14). Whether the surgical resection range can be determined according to the invasive ability of different histological types has not yet been studied. (2) RPLS usually has no specific clinical manifestation in the early stage; it is generally large-sized when discovered and easily invasive, growing in the interstitial spaces of tissues, often easily compressing, encapsulating, or invading adjacent vital organs and blood vessels. The operation is difficult, which means that residual tumor cells are more likely to be present when examined under the microscope. Moreover, due to the infiltrative extent of RPLS and the particularity of histopathology, intraoperative frozen section examination is almost impossible to achieve. With pathological advancements, a more effective method is expected to be developed for evaluating the intraoperative margin of RPLS in the future. (3) In addition, RPLS is large in size, and surgical operation often results in tumor capsule rupture. A previous study has shown that approximately 20% of tumors rupture during RPLS surgery (15), which may also be the reason for *in situ* recurrence of RPLS. RPLS often recurs near the kidney, paracolonic groove, and iliac blood vessels. The authors suggest that there may be several reasons for this: (1) There are more adipose tissues around the kidney, paracolonic groove, and iliac blood vessels. RPLS arises from the malignant transformation of adipose tissues in these regions; (2) There is relatively more soft tissue and relatively deeper infiltration of malignant cells in these areas; (3) There are relatively more important tissues and organs in these regions, which makes the operation more difficult; and (4) the probability of tumor rupture is higher, which is associated with an increased number of residual tumor cells. Future studies are expected to prove this hypothesis.

In the Transatlantic Australasian Retroperitoneal Sarcoma Working Group consensus, for patients with first local recurrence of RPS, a nomogram to predict survival is available to assist in this decision (16). Clinicopathologic variables to be considered in the decision-making process for patients with first recurrence include histologic type, grade, multifocality, and expected completeness of the second resection, among others (16). At present, extended resection mainly includes combined resection of the organs 1–2 cm around the tumor and resection of the adjacent organs without tumor invasion to ensure a negative margin. Relevant studies have shown that although extended resection can effectively reduce the recurrence rate, it is not conducive to long-term survival and reduces the quality of life of patients after surgery. There is currently no consensus on a detailed definition of extended-scope resection. The current relatively accepted surgical approach is simple and complete resection of the tumor, combined with full excision of the affected organ when organs are involved, the most common being one side of the kidney, adrenal gland, and colon (17, 18). These excisions are all aimed at improving the complete resection rate of the tumor, prolonging the survival time, and reducing the recurrence rate. According to the data obtained in this study, it remains to be further investigated whether surgical resection of the perirenal vessels, paracolonic vessels, and soft tissue near the iliac vessels, where possible, can reduce the recurrence rate of RPLS.

The most important characteristic of RPLS is that it easily recurs *in situ* after surgical resection. In this study, a total of 30 patients relapsed, with a recurrence rate of 72.2% over five years. Univariate analysis showed that Ki67 expression, tumor malignancy, and albumin level were important factors for the recurrence of the tumor after surgery (all *P* values < 0.05); the results of logistic regression analysis were consistent with the above results (*P* < 0.05). The results indicate that the differentiation degree of RPLS is closely related to the clinical features, that high-grade malignant tumors are related to local recurrence and distant metastasis, and that the pathological type and surgical margin are closely related to prognosis (19). The higher the malignancy of RPLS, the faster the growth rate of general tumors and the stronger their invasiveness. Tumors easily invade or surround the surrounding organs, blood vessels, and other tissues, which increases surgical difficulty. The higher the probability of intraoperative endoscopic tumor residue and intraoperative tumor rupture, the more likely these patients with RPLS are to have local recurrence after surgery. The more active the tumor cells are, the more likely the patients will experience recurrence. In addition, owing to the large size of retroperitoneal tumors, the nutritional status of a significant percentage of patients is deficient, as indicated by body mass index alone. A prospective study showed that 46% of patients with retroperitoneal tumors had protein malnutrition (20), with a 43.3% hypoproteinemia rate. Malnutrition may affect the production and release of inflammatory mediators, thus reducing the immune response, as well as reducing the antioxidant and direct antitumor effects (21, 22), thereby increasing the tumor recurrence rate. Ki67 is an essential tumor cell proliferation marker, which reflects the proliferative activity of tumor cells. Most studies have shown that a higher Ki67 expression represents a greater proliferative activity of tumors. Based on existing literature from China and other countries, and the current study results, we believe that the degree of RPLS malignancy, Ki67 expression positivity, and nutritional status of patients are important factors affecting postoperative recurrence. Patients with these high-risk factors for recurrence need close follow-up after surgery to reduce their risk of recurrence of RPLS and improve their prognosis. Moreover, low serum albumin may mirror a poor performance status of cancer patients who are at increased risk of death. Albumin helps to maintain intravascular oncotic pressure and acts as a radical scavenger (23). It is not known whether the association is a general oncogenic effect or attributed to a specific cancer. Previous studies have also proved that serum albumin could be used for individual risk estimation and integrated in existing prognostic models for soft tissue sarcoma (24, 25).

In this study, univariate analysis showed that tumor malignancy, Ki67 expression, and serum albumin levels were important influencing factors of the PFS time of the patient (*P* values were all < 0.05). Multivariate analysis showed that tumor malignancy and positive Ki67 expression were independent risk factors for the patient’s PFS time. Malignancy was an independent risk factor for the OS of patients with RPLS (*P* < 0.05). The 1-, 3-, and 5-year OS rates of patients in this group were 91.7%, 71.1%, and 46.6%, respectively. Recurrent episodes were the leading cause of death. Studies have shown that, compared with patients with low-grade differentiated liposarcoma, those with polymorphous liposarcoma and mixed liposarcoma with higher malignancy, high-grade differentiated liposarcoma and myxoid liposarcoma with lower malignancy had better prognoses (26). Therefore, we believe that tumor malignancy is an important factor affecting the OS of patients with RPLS. A previous study demonstrated the potential role of ECM in the mechanism of action of trabectedin in some of most frequent STS histotypes in adults. It underlines the involvement of tumor microenvironment component in predicting response to trabectedin and provide the rationale for better stratifying patients which would be candidate for this drug (27).

In the present series, the risk of local recurrence after primary resection was 2.5 percent at 3 years (28). Strategies aimed at improving outcomes for well-differentiated liposarcoma should primarily focus on enhancing the quality of surgery. The extent of surgical intervention should be tailored based on tumor location and characteristics, while minimizing the associated morbidity rate associated with extensive resection (29). It is important to consider that tumor biology may contribute to recurrence, and there could be a potential field change in the retroperitoneal and intra-abdominal fat that contributes to recurrence development (30). Future strategies to improve outcomes in dedifferentiated liposarcoma should not only concentrate on optimizing local therapy but also explore the potential of new medical treatments. A phase III multicenter randomized trial is currently underway to compare surgery alone with preoperative radiotherapy followed by surgery. The goal is to determine whether the addition of preoperative radiotherapy can reduce the risk of local recurrence. However, preliminary evidence suggests that the addition of preoperative radiotherapy may not provide significant benefits for patients with retroperitoneal sarcoma (31). Another ongoing randomized study, the STRASS 2 trial, is an international multicenter phase 3 trial that includes only high-grade dedifferentiated liposarcoma (DDLPS) and leiomyosarcoma (LMS) cases, with stratification based on specific tumor histology. The objective is to evaluate whether neoadjuvant chemotherapy can reduce the development of distant metastasis in these well-defined histological subtypes (31). This trial will be the first of its kind to include only high-grade retroperitoneal sarcoma cases and focus on two specific histological subtypes. The neoadjuvant setting offers valuable insights into tumor response *in situ*, providing opportunities for better understanding of clinical consequences, prognostic information, and research opportunities. The main limitation of our current study is that the sample size of this study is small. Moreover, HOI of the tumor has not been recorded.

In conclusion, the recurrence rate after complete resection of RPLS remains high, with the majority of recurrences located in the primary abdominal compartment. The degree of tumor malignancy, positive Ki67 expression, and serum albumin levels are important factors for the recurrence of imaging tumors. Tumor malignancy is an important factor affecting the OS of patients with RPLS undergoing complete resection. such patients should be closely followed up, with a focus on the imaging changes in the primary area of the tumor. Furthermore, the nutritional status of patients should be closely monitored to reduce the incidence of hypoproteinemia, reduce the risk of recurrence and metastasis, and improve the prognosis of patients.

## Data availability statement

The original contributions presented in the study are included in the article/supplementary material. Further inquiries can be directed to the corresponding author.

## Ethics statement

Ethical approval was not required for the study involving humans in accordance with the local legislation and institutional requirements. Written informed consent to participate in this study was not required from the participants or the participants’ legal guardians/next of kin in accordance with the national legislation and the institutional requirements.

## Author contributions

XG: Writing – review & editing. PD: Writing – original draft. ZZ: Writing – review & editing. YLi: Writing – review & editing. QZ: Writing – review & editing. DW: Writing – review & editing. XZ: Writing – review & editing. YLiu: Writing – review & editing. BT: Writing – review & editing.
